# Diversity Oriented Syntheses of Conventional Heterocycles by Smart Multi Component Reactions (MCRs) of the Last Decade

**DOI:** 10.3390/molecules17011074

**Published:** 2012-01-20

**Authors:** Heiner Eckert

**Affiliations:** Department Chemie, Technische Universität München, Lichtenbergstr. 4, Garching 85747, Germany; Email: eckert@tum.de; Tel.: +49-89-354-5532; Fax: +49-89-289-13329

**Keywords:** multicomponent reaction, MCR, MFCR, I-MCR, isocyanide, heterocycle, diversity oriented synthesis, solvent-less synthesis, alternative energy, microwave

## Abstract

A collection of smart multicomponent reactions (MCRs) with continuative post condensation cyclizations (PCCs) is presented to construct conventional three- to seven-membered heterocyclic compounds in diversity oriented syntheses (DOS). These will provide a high degree of applying economical and ecological advantages as well as of practicability. Water, ionic liquids, and solvent-less syntheses as well as use of various forms of energy as microwave and ultrasonic irradiation are examined and discussed.

## 1. Introduction

Multi Component Reaction (MCR) chemistry [1,2] applied to the synthesis of heterocycles [3,4,5] with all its variations and extensions has undergone an enormous and meaningful upturn. Seeds sown in the last century, particularly appreciated by Ugi [6], have grown enormously and provided a plurality of novel reactions, new smart strategies as well as forward-looking methods, and high product diversity [7,8]. In this paper syntheses of conventional three to seven-membered heterocyclic structures (*aziridine*, *azetidine*, *pyrrole*, *pyrrolidine*, *furan*, *indole*, *isoindoline*, *pyrazole*, *pyrazoline*, *imidazole*, *oxazolidine*, *thiazole*, *triazole*, *triazolidine*, *tetrazole*, *pyridine*, *pyrane*, *isoquinoline*, *pyrimidine*, *piperazine*, *oxazine*, *tetrazine*, and *oxadiazepine*) are presented. The heterocycles are listed in see Section 2), in ascending order, firstly by ring-size 3 to 7, secondly by number of hetero atoms 1 to 4, and thirdly by hetero atoms N, O, S.

### 1.1. Times and Progress

**Scheme 1:**
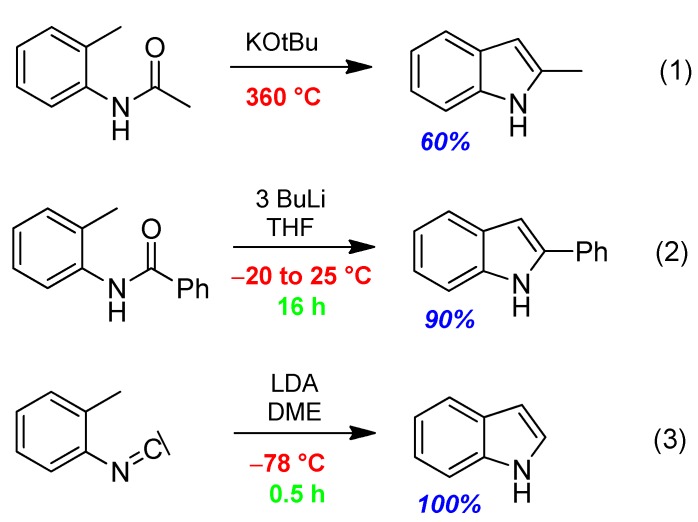
Indole syntheses (**1**)–(**3**) under various reaction conditions.

First of all, three syntheses of indole/indole derivatives, according to (1) [9], (2) [10], and (3) [11] in Scheme 1, shall demonstrate the course of time and progress in research, how reactions and their conditions became better in terms of ecology and economy, *i.e.*, reaction temperatures fell from 360 °C to r.t. (room temperature) and −78 °C, reaction times decreased from 16 h to 30 min and product yields increased from 60% to 100% (Table 1). 

To compare data of not exactly identical products is problematic, but the principal trend in the different data is evident.

**Table 1:** Reaction data of indole syntheses (**1**)–(**3**).

Reaction-conditions	Madelung Synth. (1)	Modified Madelung (2)	Saegusa Synth. (3)
Publ. year	1912	1981	1977
Base	KOtBu	BuLi	LDA
Temp [°C]	360	−20 to +25	−78
Time [h]	n.a.	16	0.5
Yield [%]	60	90	100

Beyond this, (3) shows, that using an isocyanide function the reaction becomes faster. This advantage of highly reactive isocyanide reagents is also evident for isocyanide-based MCRs (I-MCRs) such as the Passerini- and Ugi-reactions [6] as well as novel I-MCRs presented in see sub-section 3.2.

### 1.2. Nomenclature

Due to the rapid progression of MCRs by processing additional functions and its extensions into domino- and post-condensation-cyclisations (PCCs) (sub-section 3.4 and sub-section 3.5), the hitherto existing nomenclature is no longer sufficient for many cases and becomes sometimes unwieldy and imprecise. A consequence of these impacts is a different counting of components and active functions. The latter can be emcompassed within the phrase “Multi-Function-Component-Reaction” or “MFCR”, respectively [2], and is so used in this article. *i.e.*, when the number of functions in the reaction involved exceeds the number of components, the former will be prefixed to the latter. For example, U-5F4CR means an Ugi four component reaction with five participating functions, as is in following  sub-section 2.1. Often the extension of functions will cause a domino-reaction.

## 2. High Diversity in Heterocycle Syntheses with MCRs

Nowadays nearly all heterocycles can be constructed using MCRs. Here, a survey of recent developments on Diversity Oriented Syntheses (DOS) will be reported [7,8]. Diversity of products is increasing by both versatile and smart MCRs and many consecutive further reactions like versatile domino-reactions and post-condensation-cyclisations (PCCs) [12,13]. This can also be achieved by an increase of the number of components, as in 5CR [14], 7CR [15], and 8CR [16], transition metal catalysed MCRs [17], and evolutionary chemistry aided MCRs [18]. Several recent diversity oriented reviews [19,20,21,22,23,24,25,26] demonstrate the high innovation and creativity in this seminal field of chemistry.

### 2.1. Aziridine **3**, U-5F4CR, 2-Alkoxyketone + Carboxylic acid + Amine + Isocyanide [27]

**Scheme 2:**
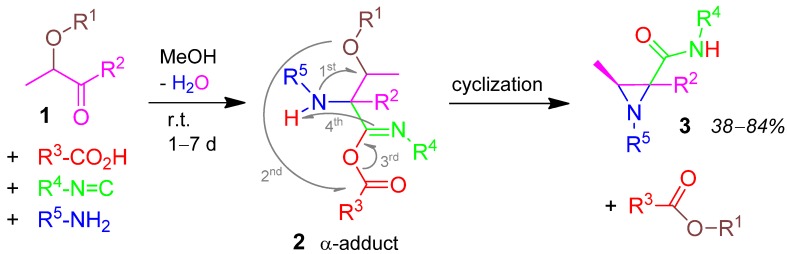
Synthesis of aziridine **3** by U-5F4CR.

Compound **1** is the bi-functional carbonyl component in the Ugi-four component reaction (U-4CR) in addition to carboxylic acid, isocyanide, and primary amine. After forming the α-adduct **2**, the generated *sec.* amine substitutes the vicinal alkoxy-group (formerly the second function of **1**). The latter reacts with the acyl function of the aza-anhydride moiety forming the *carboxylic acid ester* by-product. The Ugi-reaction goes on to form the target molecule, the tetra-substituted aziridine derivative **3** (Scheme 2). Yields are moderate to good (38–84%) [27].

Further syntheses of aziridines and oxiranes are of 2-aminoketones from natural α-amino acids [28], and S-ylide-mediated 3CR of alkylsulfonium salts with imines or aldehydes [29].

### 2.2. Azetidinone **4**, U-4F3CR, β-amino Acid + Aldehyde + Isocyanide [30]

**Scheme 3:**
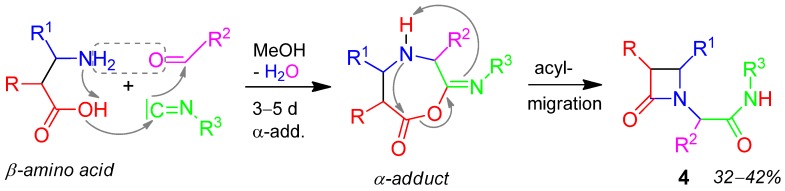
Synthesis of azetidinone **4** by U-4F3CR.

Tri-substituted azetidinone **4** is formed easily from β-amino acids by an Ugi-4F3CR in 32–42% yield (Scheme 3) [30]. Thereby two functions amino- and carboxylic acid-groups are located in one component β-amino acid. A review on β-lactam antibiotics syntheses by similar MCRs has been published [31].

### 2.3. Azetidine **6**, 3CR, Azabicyclo[1.1.0]butane + 2,3-Dicyanofumarat + Alcohol [32]

**Scheme 4:**
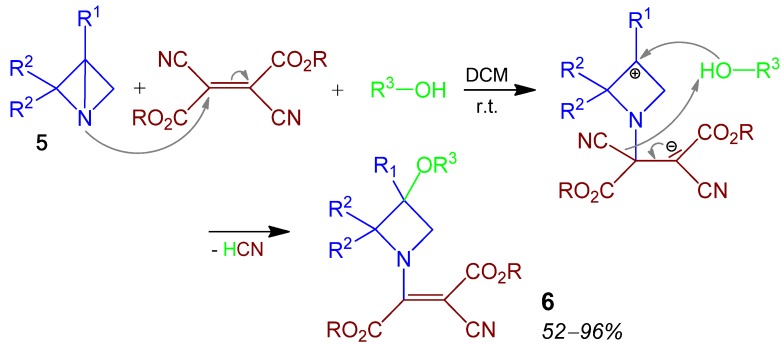
Synthesis of azetidine **6** by 3CR.

The azabicyclo[1.1.0]butane building block **5**, recently used on various occasions, reacts in a 3CR with Michael-acceptor 2,3-dicyanofumarate and alcohols to form pentasubstituted azetidines **6** in moderate to excellent yields of 52–96% (Scheme 4) [32].

### 2.4. Pyrrole **7**, H-4F3CR, 2-Ketoester + Amine + Fumaric Dichloride [33]

**Scheme 5:**
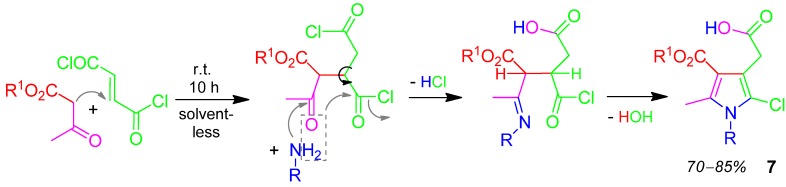
Synthesis of pyrrole **7** by H-4F3CR.

This Hantzsch-4F3CR forms pentasubstituted pyrrole derivatives **7** and runs solvent-less at r.t. to provide good yields (70–85%) of **7** [33]. Thereby fumaric dichloride functions as Michael acceptor for the addition of the acetoacetate. Eliminating water transforms one acyl chloride into a carboxylic acid function (Scheme 5).

Another *solvent-less* synthesis of pyrrole from diazadiene + acetoacetate + amine has been performed with good yields of 56–84% [34].

### 2.5. Pyrrole **9**, H-3CR, Acetylenedicarboxylate + Diacetyl + Ammonium Acetate [35]

**Scheme 6:**
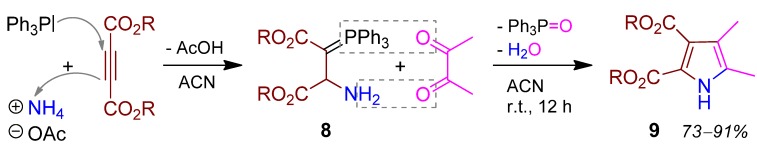
Synthesis of pyrrole **9** by H-3CR.

The deactivated alkyne dialkyl acetylenedicarboxylate is activated by Ph_3_P and reacts with the ammonium cation at r.t. to form the aminophosphorane **8**. This undergoes a Wittig reaction with diacetyl and reacts further according to Hantzsch-3CR, affording the 2,3-dimethyl.4,5-dialkoxy-carbonyl substituted pyrrole derivative **9** (Scheme 6) [35].

### 2.6. Pyrrolidine 11, I-4CR, Aldehyde + Malodinitrile + Isocyanide + Phenanthridine [36]

**Scheme 7:**
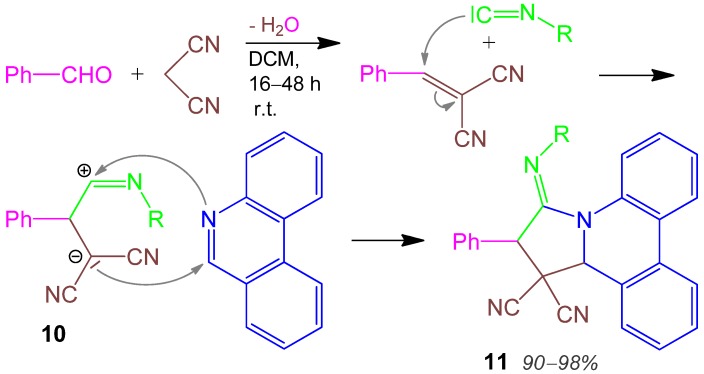
Synthesis of pyrrolidine **11** by I-4CR.

First part of the I-4CR is a Knoevenagel condensation of an aromatic aldehyde with malodinitrile. This intermediate reacts with an isocyanide to form a reactive ylide **10**, which adds to the azomethine function of phenanthridine, furnishing the phenanthridopyrrolidine **11** in very good to excellent yields of 90–98% (Scheme 7) [36]. Elimination of HCN does not occur.

Further syntheses are a highly diastereoselective 3CR from aldehyde + aminomalonate + nitro-alkene to form 2,3,4,5-tetrasubstituted pyrrolidines [37], a chiral ruthenium porphyrin-catalyzed 3CR with chiral azomethine ylides [38], 4CRs of aldimines + acyl chlorides + alkynes + CO [39], and a reaction of alkyne + *p*-tolylsulfonylmethyl isocyanide (TosMIC) [40]. Two 3CRs with aniline + glyoxylate + bromobenzophenone [41] and diazoketone + nitroalkene + amine [42] have been performed. An extensive and critical review on the use of MCRs to synthesize pyrrole compounds has been published [43].

### 2.7. Amino-furan **13**, I-3CR, Acetylenedicarboxylate + Acid + Isocyanide [44]

**Scheme 8:**
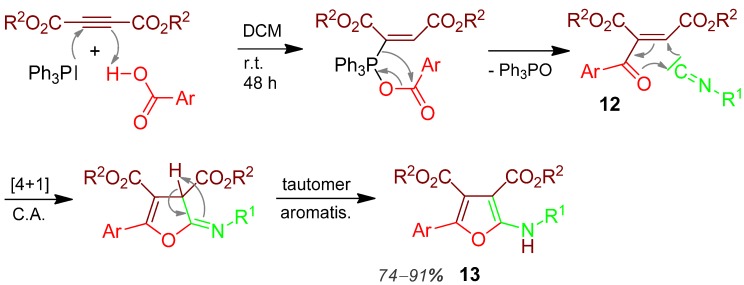
Synthesis of amino-furan **13** by I-3CR.

In an I-3CR the deactivated alkyne dialkyl acetylenedicarboxylate is activated by (Ph)_3_P, which also deoxygenates the carboxylic acid acyl group, generating the 1,4-dipole **12**, which reacts with the isocyanide in a [4+1] cycloaddition reaction. Final aromatisation by thermodynamically driven tautomerisation affords the tetrasubstituted aminofuran derivative **13** in good yields of 74–91% [44] (Scheme 8).

Further furan syntheses are a 3CR of salicylaldehyde + amine + alkyne, [45] and a 3CR performed with an imidazolium salt + alkyne + aldehyde [46].

### 2.8. Indole **15**, 3CR, Haloarylketone + Sulfoniumylide + Amine [47]

**Scheme 9:**
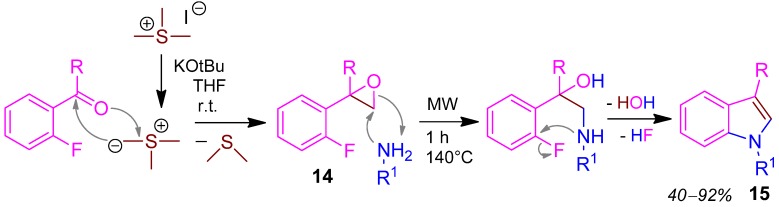
Synthesis of indole **15** by Corey-Chaykovsky 3CR.

The reaction of the ketone with the sulfonium ylide forms the epoxide intermediate **14** by elimination of dimethylsulfide. Compound **14** reacts with a primary amine in a microwave assisted Corey-Chaykovsky reaction affording the indole derivative **15** in 40–92% yield (Scheme 9) [47]. Last step is the thermodynamically driven aromatisation by β-elimination of water.

Further indole syntheses by multi-component reactions are a new 3CR Fischer indole syntheses based on new reactions of organometallic reagents with nitriles and carboxylic acids, which extend scope and synthetic utility of these syntheses [48], MCR of indoles from 2-iodobenzoic acid [49], and highly diversified indole scaffolds by U-4CR [50].

### 2.9. Pyrazole **17**, I-3CR, Acetylenedicarboxylate + Isocyanide + Semicarbazide [51]

**Scheme 10:**
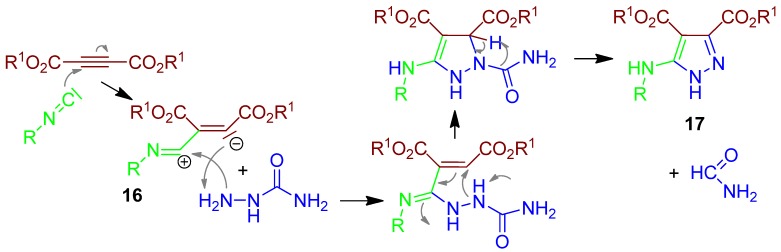
Synthesis of pyrazol **17** by I-3CR.

The isocyanide reacts in an I-3CR with the deactivated alkyne dialkyl acetylenedicarboxylate to form a reactive dipole intermediate **16**, which reacts with the hydrazine moiety of semicarbazide. In the last step formamide is eliminated as a by-product, providing the aromatic 3,4,5-trisubstituted pyrazole derivative **17** (Scheme 10) [51]. 

A new and environmentally-friendly method for preparing dihydropyrano[2,3-c]pyrazoles in water as solvent and underultrasound irradiation has been developed [52].

### 2.10. Pyrazoline **19**, 3CR / PCC, Cyclopropylketone + Amine + Aldehyde / + Hydrazine [53]

**Scheme 11:**
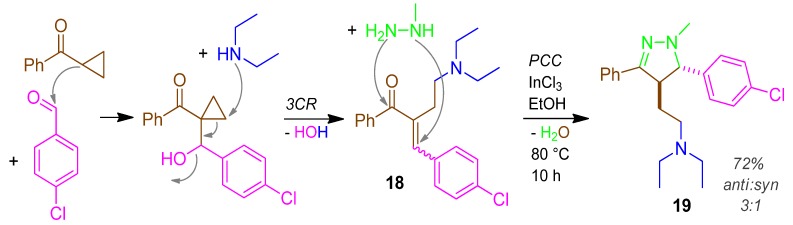
Synthesis of pyrazoline **19** by 3CR/PCC.

Cyclopropylphenylketone and *p*-chlorobenzaldehyde react to form the aldol addition product. This undergoes with diethylamine β-elimination of H_2_O affording the 3-CR product Michael acceptor **18**. Methylhydrazine reacts regioselectively with **18** according to the HSAB-concept in a PCC forming the pyrazoline derivative **19** with 72% yield and an *anti/syn* ratio of 3 (Scheme 11) [53].

### 2.11. Imidazole **21**, U-5F4CR / PCC, Acid + Amine + 2-Ketoaldehyde + Isocyanide / + NH_3_ [54]

**Scheme 12:**
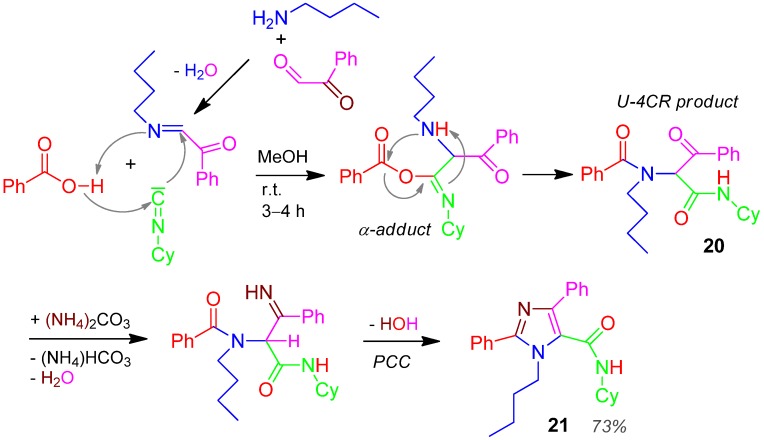
Synthesis of imidazole **21** by U-5F4CR/PCC.

The U-4CR of benzoic acid, *n*-butylamine, 2-ketophenylacetaldehyde, and cyclohexylisocyanide provides **20**, which reacts with ammonia (from ammonium carbonate) in a PCC cyclic amidination reaction to form the 1,2,3,5-tetrasubstituted imidazole **21**, yield is 73% (Scheme 12) [54].

Further syntheses of imidazoles are MCRs from 1,2-diketone + aryl aldehyde + amine + NH_3_ [55] and from aziridine + alkyne + TsN_3_ [56]. TosMIC-based 3CRs form 4,5-disubstituted imidazole derivatives in good yields of 62–86% [57]. 4CRs from benzil + benzaldehyde + aniline + ammonium acetate in the ionic liquid butylmethyl imidazolium bromide as solvent [58] or solvent-free with “solid carbon acid” as catalyst [59] and both conventional or microwave-assisted*,* provide tetraaryl-substituted imidazoles both in very good yields. 

### 2.12. Imidazolium Salt **25**, I-3CR, N-methyldihydropyridin + 2 x Isocyanide + Iodine [60]

**Scheme 13:**
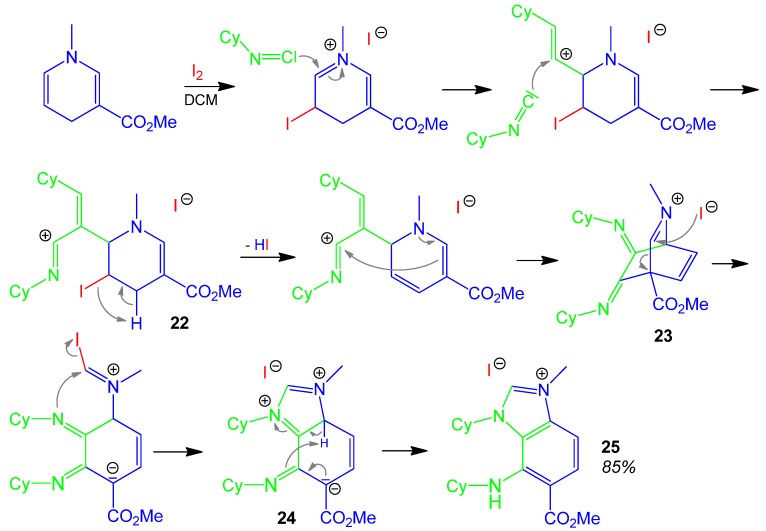
Synthesis of imidazolium salt **25** by I-3CR.

A direct access to benzimidazolium salts has been achieved by a 3CR with *N*-methyl-dihydropyridine-3-carboxylate, two cyclohexyl isocyanides and iodine. The proposed reaction mechanism is rather complex. Core steps of the reaction are the double α-addition of isocyanides forming **22**, the rearrangement of the dihydropyridine into the aza-bicyclo[2.2.2]octadiene structure **23**. Ring-opening of **23** and ring-closure furnish the benzimidazolic zwittwerionic structure **24**. Full aromatisation of **24** is thermodynamically driven and affords finally the benzimidazolium iodide **25** in a high yield of 85% (Scheme 13) [60]. 

A similar approach to create a mesoionic structure has been performed by a 3CR from isocyanide + isochinoline + trifuoroacetic anhydride (TFAA) forming an isochinolinoimidazoliumylide [61].

### 2.13. Oxazolidinone **27**, 3CR, Aldehyde + Amine + Alkyne + CO_2_ [62]

**Scheme 14:**
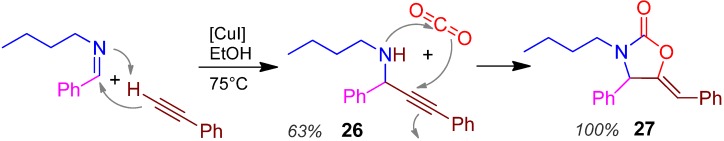
Synthesis of oxazolidinone **27** by 3CR.

After condensation of aldehyde and amine to the imine, this reacts with the alkyne (CuI-catalyzed) to form **26**. This cyclizes with carbon dioxide to afford the 3,4,5-trisubstituted 1,3-oxazolidin-2-one **27** quantitatively [62]; the overall yield is 63% (Scheme 14).

A 3CR from isocyanoacetate + aldehyde + amine provides trisubstituted oxazoles in good yields of up to 96% [63].

### 2.14. Thiazole **30**, Domino U-4CR / PCC, Thioacid + Amine + Isocyanide + Aldehyde [64]

**Scheme 15:**
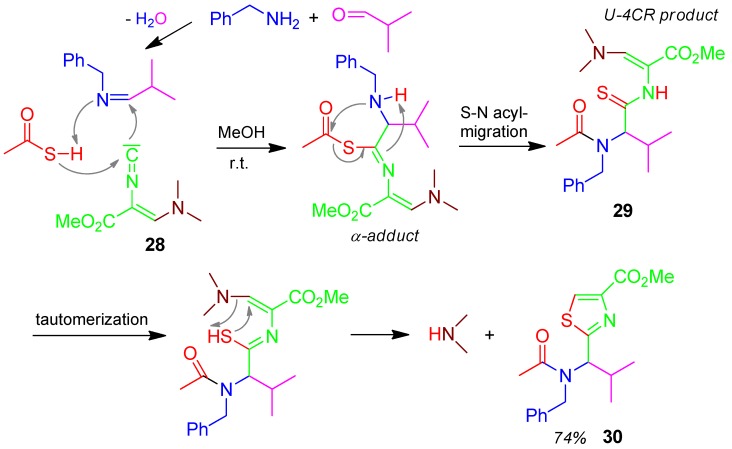
Synthesis of thiazole **30** by U-4CR/PCC.

A U-4CR reaction effects the transfer of the thiol group onto the isocyanide carbon atom. After tautomerization of the thio group in the Ugi product **29** into a thiol function, this adds to the Michael acceptor of the former Schöllkopf isocyanide **28** to form the 2,4-disubstituted thiazole derivative **30** in 74% yield *via* elimination of dimethylamine (Scheme 15) [64].

A further 2,4,5-trisubstituted thiazole domino U-4CR/PCC is prepared from oxo-components + primary amines + thiocarboxylic acids + methyl 3-bromo-2-isocyanoacrylates [65]. 

### 2.15. Oxazino-1,2,3-triazole **33**, Domino P-5F3CR / PCC [3 + 2], Propiolic Acid + Isocyanide + Azido-aldehyde [66]

**Scheme 16:**
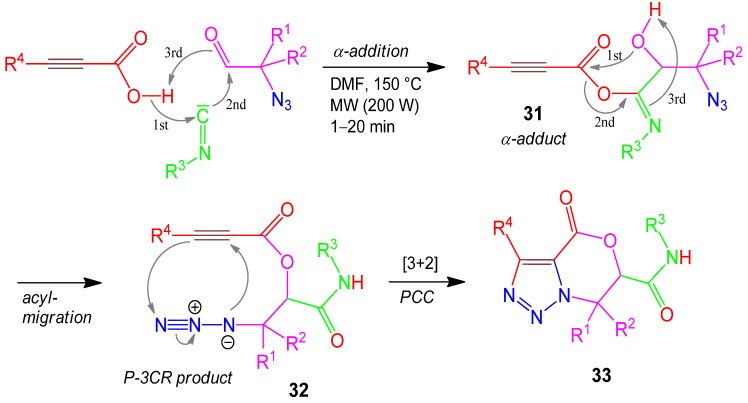
Synthesis of oxazino-1,2,3-triazole **33** by P-5F3CR/PCC.

In a MW-assisted Passerini-3CR propiolic acid and azidoaldehyde added to an isocyanide (α-addition) affording the α-adduct **31**. The acyl group of the iminoester moiety migrates to the alcohol function of the former aldehyde moiety (acyl migration) to furnish the P-3CR product **32**. The two additional acetylene and azide functions now react in a [3+2] cycloaddition forming the 1,2,3-triazole moiety of the final product **33** (Scheme 16). Azidoaldehydes are rather instable compounds, so they are employed in the synthesis as their corresponding azidoalcohols, which are oxidized by IBX directly before starting the P-3CR. Yields of **33** based on the azidoalcohols are 62–70% [66].

Similar syntheses using U-4CRs instead of the P-3CR of before have also been performed [67]. Not a MCR, but a highly efficient Cu(I)-isocyanide complex has been developed as a heterogeneous catalyst for azide-alkyne cycloaddition in water [68] to improve click chemistry (CC) [69,70,71].

### 2.16. 1,2,4-Triazolidine **35**, 3CR, Azodidicarboxylate + Imine + Alkyl Diazoacetate [72]

**Scheme 17:**
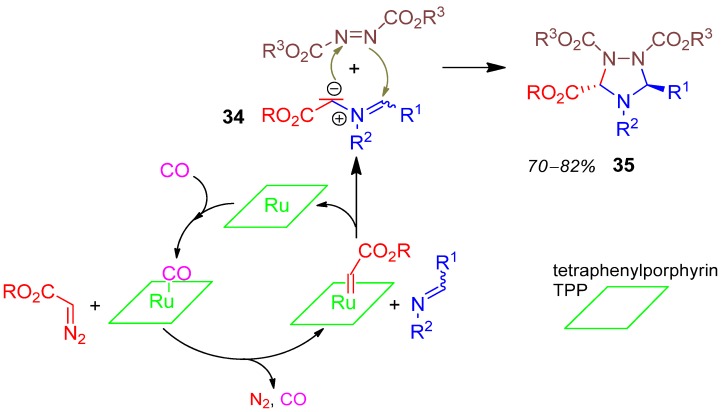
Synthesis of 1,2,4-triazolidine **35** by 3CR.

The 3CR of alkyl diazoacetate with an imine is catalyzed by ruthenium tetraphenylporphyrin (RuTPP) accompanied by the release of nitrogen and forming the strong 1,3-dipole azomethinylide **34**, which reacts with the dialkyl azodicarboxylate to furnish in a stereocontrolled [3 + 2] cycloaddition reaction the pentasubstituted 1,2,4-triazolidine **35** in 70–82% yield (Scheme 17) [72].

A general method for the synthesis of 1,3,5-trisubstituted 1,2,4-triazoles has been developed from 3CR of carboxylic acid + primary amidine + monosubstituted hydrazine [73]. This highly regioselec-tive one-pot process provides high diversity and yields up to 78%.

### 2.17. Tetrazole **37**, 3CR, Formic Acid Orthoester + Amine + Azide [74]

**Scheme 18:**
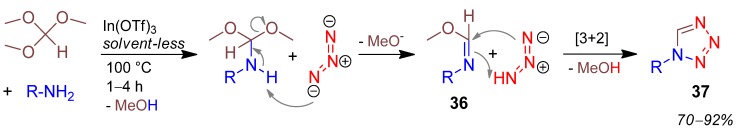
Synthesis of tetrazole **37** by 3CR.

Trimethyl orthoformate and amines react to form **36**, which cyclise with HN_3_ to form the tetrazoles **37** in good yields of 70–92%. The reaction runs without any solvent (Scheme 18) [74].

### 2.18. Tetrazolyl Isoindoline **40**, Domino U-5F4CR / PCC, 2 x Cyclization Reaction, Methyl Formyl-benzoate + HN_3_ + Amine + Isocyanide [75]

**Scheme 19:**
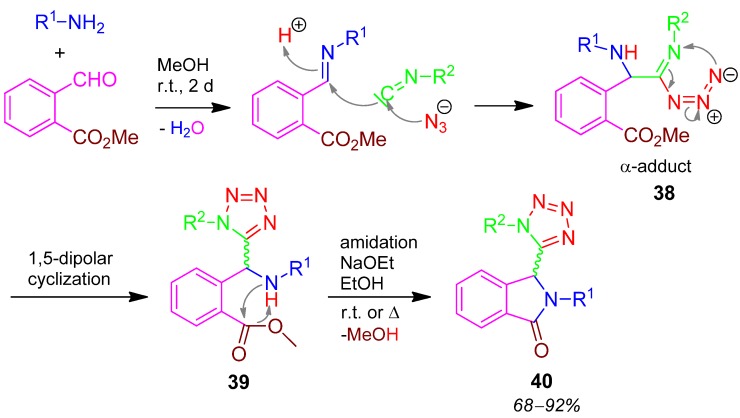
Synthesis of tetrazolyl isoindoline **40** by U-5F4CR.

In an U-4CR with HN_3_ as acidic component the α-adduct **38** is formed and a 1,5-dipolar cyclization takes place affording the tetrazole **39**. A PCC by an amide generation furnishes the isoindoline in the final product **40** with yields of 68–92% (Scheme 19) [75]. I-3CR with isocyanide + bromine + azide provides 5-bromotetrazole quantitatively [76]. The bromo function can further react in a Suzuki-reaction giving a 97% yield of the subsequent product.

### 2.19. Pyridine **45**, Domino U-5F4CR / PCC, 2-Ketoacid + Amine + Aldehyde + Isocyanide [77]

**Scheme 20:**
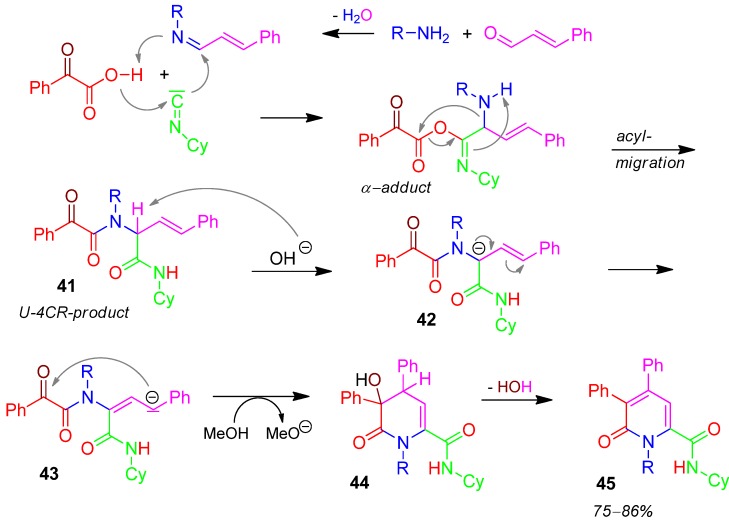
Synthesis of pyridine **45** by U-5F4CR.

After reaction of 2-ketocarboxylic acid, amine, aldehyde, and isocyanide the resulting U-4CR-product **41** is deprotonated by hydroxide anion to form the strong carbanion **42**, which isomerizes to give **43**. 1,6-Cyclization of **43** provides **44**, which undergoes aromatization by dehydration to form the pyridine derivative **45** in good yields of 75 to 86% (Scheme 20) [77].

In a MW-assisted 3CR in water as solvent, isoxazolopyridines have been prepared in short reaction times of 6–9 min at temperatures of 100–130 °C and in very good yields of 84–94% [78]. Solvent-less 4CR of acetylenedicarboxylate + malonitrile + amine + aldehyde provides pentasubstituted 2-amino-pyridines in very good yields of 79–95% [79]. A 3CR of pyrimidine + indole + aminoacryl-nitrile in water as solvent furnishes pyrazopyridine in a complex structure in high yields of 75–95% [80]. Pyrazopyridines have also been synthesized in a 3CR in the IL butylmethyl imidazolium bromide or tetrafluoroborate as solvent in good yields of 79–95% [81,82].

Syntheses of pyrazolopyridines have been performed by 3CR from aldehyde + malodinitrile + aminopyrazole activated with both conventional heat (in refluxing ethanol) and ultrasound (45 kHz, 305 W, 60 °C) [83]. Yields have been 50–65% in conventional thermal and 85–98% in ultrasound driven reactions for the same ten products.

### 2.20. Pyran **47**, Domino I-3F3CR/PCC, Acetylenedicarboxylate + Hydroxynaphthoquinone + Isocyanide [84]

**Scheme 21:**
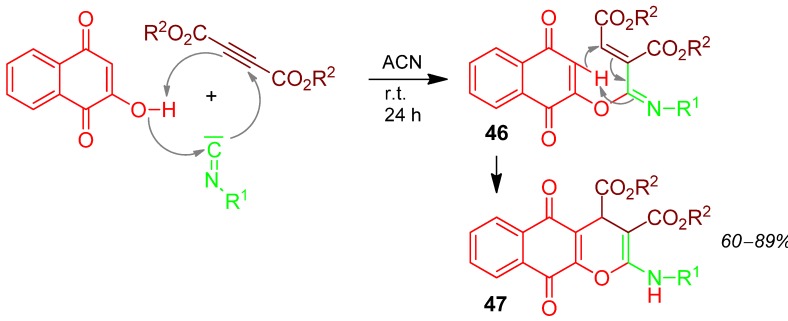
Synthesis of pyrane **47** by I-3CR/PCC.

Hydroxynaphthoquinone, acetylenedicarboxylate, and isocyanide react in an I-3CR to give **46**, which cyclises to form the pyran derivative **47** in good yields of 60–89% (Scheme 21) [84].

### 2.21. Isoquinoline **50**, 5F4CR, Alkynylbenzaldehyde + Primary Amine + Formaldehyde + Secondary Amine [85]

**Scheme 22:**
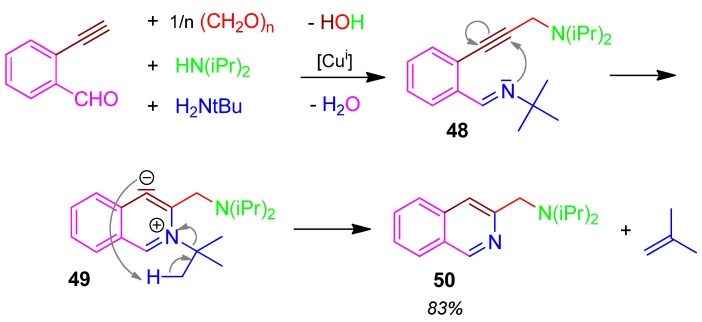
Synthesis of isoquinoline **50** by 5F4CR.

The Cu(I) iodide-catalyzed 5F4CR of 2-ethynylbenzaldehyde, paraformaldehyde, diisopropylamine and *tert*.-butylamine affords **48**, which further reacts to form a strong carbanion **49**. Its protonation provides the 2-aminomethylisochinoline **50** under elimination of isobutene as by-product [85] (Scheme 22).

### 2.22. Pyridopyrimidine **52**, I-4F3CR, 2-Aminopyridine + Acetylenedicarboxylate + Isocyanide [86]

**Scheme 23:**
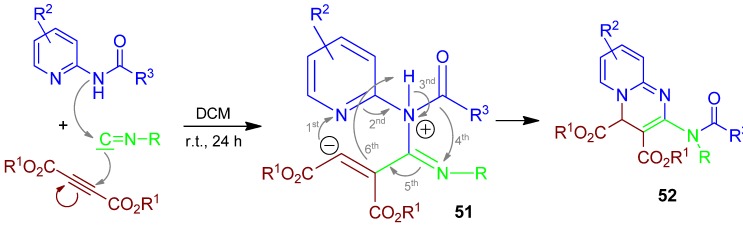
Synthesis of pyridopyrimidine **52** by I-4F3CR.

Isocyanide-based 4F3CR from 2-aminopyridine, deactivated alkyne acetylenedicarboxylate, and isocyanide forms the zwitterionic compound **51**, which reacts to yield the pyridopyrimidine **52** [86] (Scheme 23).

2-Amino-5-cyano-pyrimidine-6-one has been synthesized by a 3CR of aldehyde + cyanoacetamide + guanidine [87]. An easy access for imidazopyrimidine derivatives from aldehyde + 1,3-diketone + 2-aminobenzimidazole with good yields of 60–82% is presented in [88].

### 2.23. Piperazine **55**, U-4F3C, Ketocarboxylic Acid + Amine + Isocyanide [89]

**Scheme 24:**
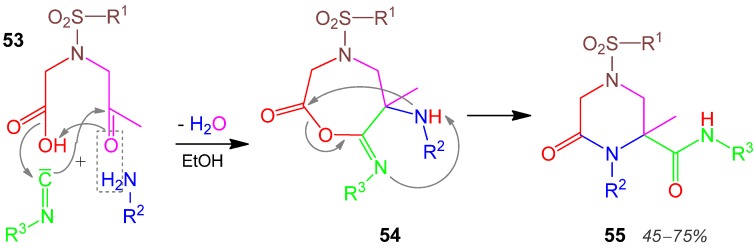
Synthesis of piperazine **55** by U-4F3CR.

In an Ugi reaction with both carboxylic acid and aldehyde functions connected in one component **53** with a neutral additional sulfonamide moiety, **53** reacts with an amine and an isocyanide to give the α-adduct **54**. After the acyl-migration at **54** the 1,4,6-tetrasubstituted piperazinone-derivative **55** is formed in 45–75% yield (Scheme 24) [89]. A diversity-oriented synthesis (DOS) of a piperazine library with 90 analogs have been performed by use of MCRs [90]. A review on piperazine syntheses by MCRs has been published recently [91].

### 2.24. Tetrazinane **57**, 3CR, Aldehyde + Urea + Ammonia [92]

**Scheme 25:**
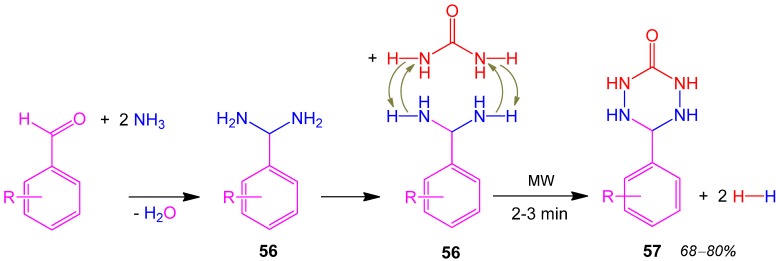
Synthesis of tetrazinane **57** by 3CR.

In a 3CR benzaldehyde aminoacetal **56** is formed. A sequence of metathesis reactions connect the amino functions of both **56** and urea together forming 6-aryl-1,2,4,5-tetrazinane-3-one **57** in good yields of 68–80% [92]. Two equivalents of hydrogen are released (Scheme 25). 

The proposed mechanism is favored by the lack of any solvent, so that N-H moieties are in direct contact with each other and are strongly activated by microwave irradiation (MW). A strong evidence for the proposed mechanism is given by comparing the data of the MW-supported reaction with the conventionally heated reaction. The MW-supported reaction rate is 15 fold and the product yield twice that of the conventionally heated process.

### 2.25. Oxadiazepine **59**, U-6F4CR / PCC Staudinger-aza-Wittig reaction, Azidocarboxylic Acid + Aldehyde + Isocyanide + Aminoketone [93]

**Scheme 26:**
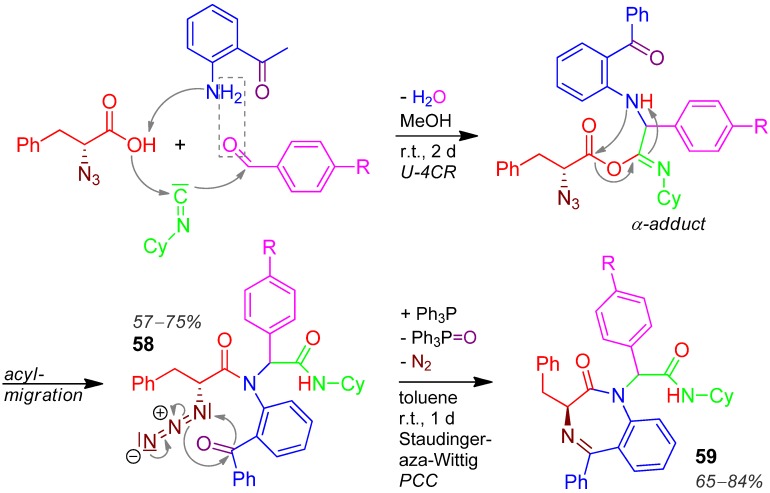
Synthesis of oxadiazepine **59** by U-6F4CR/PCC.

In an U-4CR wherein two components with two functions each, 2-aminobenzophenone, 2-azido-3-phenylpropionic acid, benzaldehyde, and cyclohexyl isocyanide react forming the Ugi product **58** in 57–75% yield. A Staudinger-aza-Wittig sequence effects the ring closure furnishing the (*S*)-3-benzyl-2-oxo-1,4-benzodiazepines **59**, yields are 65–84% (Scheme 26) [93]. The ketone carbonyl oxygen atom is removed by triphenylphosphane.

### 2.26. Oxadiazepine **62**, Domino Aza-Wittig / I-3CR, Acetylenedicarboxylate + 1,3-Diketone + Isocyano-azaphosphorane [94]

**Scheme 27:**
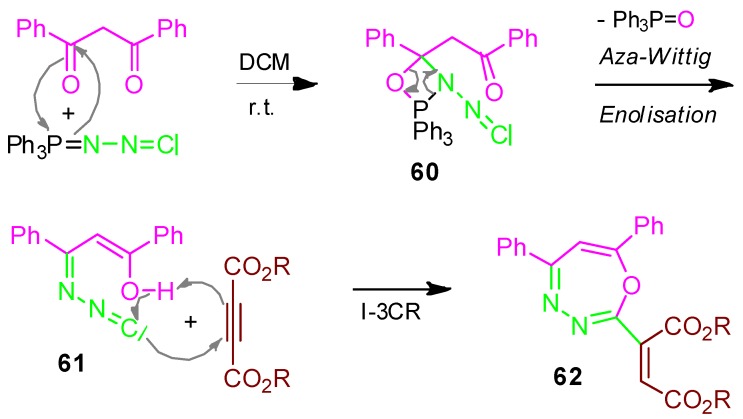
Synthesis of oxadiazepine **62** by I-3CR.

The aza-Wittig-reaction of a 1,3-diketone with an isocyanoazaphosphorane provides the phospha-oxazetidine derivative **60**, which enolises to give the isocyanide **61**. This further reacts with dialkyl acetylenedicarboxylate to form the 1-oxa-3.4-diazepine derivative **62** (Scheme 27) [94].

## 3. Strategies in Designing Novel MCRs

Several thermodynamic and reaction chemical effects as well as new strategies and new developed synthetic methods are smart and very advantageous ways to create novel MCRs.

### 3.1. Thermodynamic Effects

The well-known strategy to effect an easy forming of heterocyclic compounds by aromatising them has been often applied in syntheses, as illustrated by almost half the reactions in this paper. There, a resonance energy in the range of 100 kJ/mol is released. Particularly in the last step of a synthesis, the exergonic behavior makes this an irreversible one and affords good product yields. This is demonstrated by reactions with tautomerisations as the last step in sub-sections 2.7 and 2.20.

Another strategy to facilitate a reaction course is to accelerate the number of molecules on the product site of the syntheses. Thus, reaction entropy increases and facilitates cyclization. This can be done by introduction of appropriate substructures into the reaction paths, which will later become leaving groups to push the progress of the reactions, preferably in the last step. These are H_2_O in sub-sections 2.8, 2.11 and 2.19, MeOH in 2.17, N_2_ in 2.16., CO_2_ in 3.1.1, carboxylic acid ester in 2.1, dimethyl sulfide in 2.8, formamide in 2.9, dimethylamine in 2.14, isobutene in 2.21, isocyanate in 3.1.1, and even ketene in 3.1.2. The latter two by-products are somewhat unusual as leaving groups, so their reaction pathways will be discussed. 

#### 3.1.1. Isocyanate **63** Elimination [95]

**Scheme 28:**
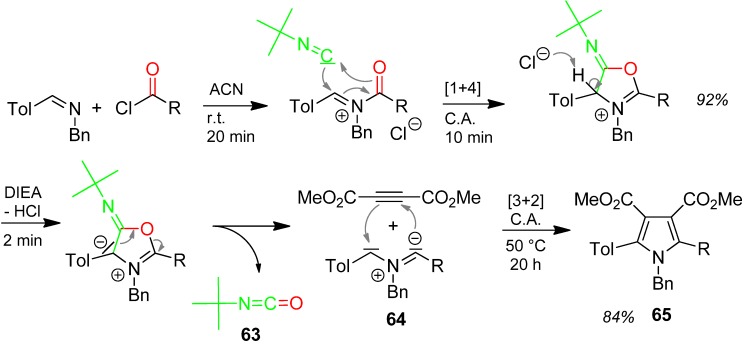
Isocyanate **63** formation as by-product in a pyrrole synthesis.

The reaction is mediated by *tert*.-butyl isocyanide. By-product of the pyrrole derivative synthesis is *tert*.-butyl isocyanate **63**, which comes from the oxygenation of *tert*.-butyl isocyanide with oxygen from the acyl moiety (Scheme 28), which leaves back the strong azomethineylide 1,3-dipole **64**. This reacts with the acetylenedicarboxylate in a [3+2] cycloaddition affording the pentasubstituted pyrrole derivative **65** in good yield of 84% [95].

A highly interesting comparison of the same pyrrole synthesis by a 4F3CR catalyzed by Pd(0), but mediated by CO instead of isocyanide has been published in the same article [95]. In this case, CO_2_ is formed by the oxygenation of CO.

#### 3.1.2. Ketene **68** Elimination [96]

**Scheme 29:**
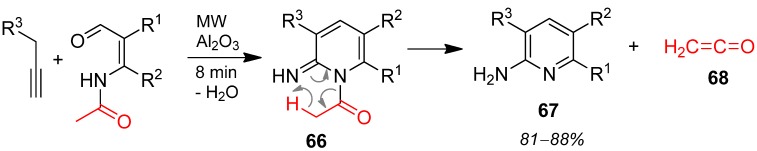
Ketene **68** formation as by-product in a pyridine synthesis.

Nitrile and acylaminoketone react MW-assisted and fast to form **66**. After eliminating the acyl residue the 3,5,6-trisubstituted 2-aminopyridine derivative **67** is formed in good yields. The side product of the synthesis is the high-energy-molecule ketene **68**, which has been generated in an electrocyclic reaction of **63** and will further react (Scheme 29) [96].

### 3.2. Isocyanide-Based MCRs (I-MCRs)

Various types of reactions have been presented and the corresponding reaction mechanisms described for each reaction in Section 2. Most applied functional groups in MCRs are also usual reaction partners in general chemical reactions and some reviews [97,98,99,100,101,102] have been published on the use of malodinitriles in MCRs [97], 1,3-dicarbonyl building blocks [98,99], aldehydes and β-ketoesters for Biginelli reactions [100], acetylenedicarboxylates [101], and imine-based MCRs [102].

**Scheme 30:**
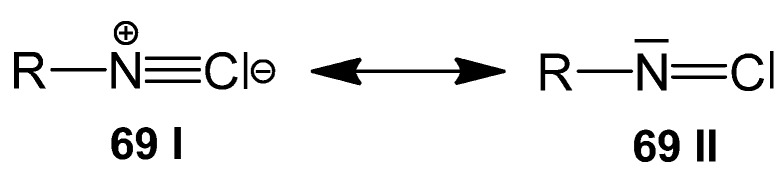
Resonance effect of isocyanide **69**.

Isocyanides **69**, however, are typical MCR components [6,103,104,105,106,107,108,109], and half of the reactions presented in this paper are based on isocyanides. Within the last decade, about a thousand papers on I-MCRs have been published. This outstanding position is the consequence of isocyanides’ electronic structure as electron-rich carbenoids (Scheme 30). Whereas mesomer **69 I** emphasises the nucleophiliccharacter of the isocyanide, mesomer **69 II** demonstrates the carbene nature of the isocyanide with its electron deficient sextet at the C-atom. This makes an isocyanide favored to react with a nucleophile *and* an electrophile simultaneously in an α-addition reaction, the I-3CR (as is P-3CR in sub-section 2.15). Thereby the transition of the divalent carbon C^II^ of the carbenoid isocyanide into the sp^2^-hybridised tetravalent C^IV^ of the α-adduct of the isocyanide with two reaction partners takes place. This is the irreversible step in the reaction of isocyanide-based MCRs and drives the reaction course to the product side.

Since the pioneering and seminal research on isocyanide-based MCRs by Passerini and Ugi, who recognized their enormous potential and made this chemistry presentable, many extensions and some new I-MCRs have been created. Outstanding and recently intensively researched reactions are the combination of isocyanides with electron-deficient alkynes as are dialkyl acetylenedicarboxylates, generating the reactive zwitterionic intermediate (as **16** in sub-section 2.9), which could be trapped by a third component [108,109]. Several syntheses of this type are presented in sub-section 2.7 [44], 2.9 [51], 2.20 [84], 2.22 [86], and 2.26 [94] of this paper.

Another important progress is the I-4CR from aldehyde + malodinitrile + imine + isocyanide [97]. Aldehyde and malodinitrile react in a Knoevenagel condensation forming a strong Michael acceptor, which adds as well as the imine to the isocyanide, according to the reaction in sub-section 2.6 [36], and 2.19 [79,83]. A reactive ylide-intermediate makes the reaction definite, achieving high product yields.

### 3.3. Isocyanide Generation

**Scheme 31:**
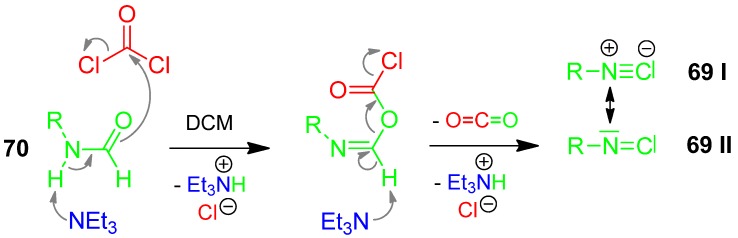
Generation of isocyanides from formamides.

The general and common generation of isocyanides **69** is still the dehydration of the corresponding formamides **70** by dehydrating reagents such as phosgene (Scheme 31), diphosgene, triphosgene, chloroformates, phosphorous chlorides, sulfonyl chlorides, the Appel reagent (triphenylphosphine/ haloalkanes), CDC (2-chloro-1,3-dimethylimidazolium chloride), and the Burgess reagent (methyl carboxysulfamoyl-triethylammonium ylide) [110]. A solvent-free and safe process to produce phosgene from solid triphosgene in amounts ranging from grams to kilograms is given [111,112].

**Scheme 32:**
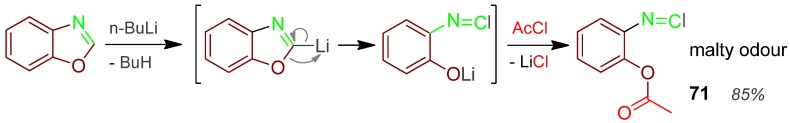
Generation of isocyanides from 1,3-oxazoles.

A new trend may sometimes help to solve an old problem, namely how to handle the disgusting odour of isocyanides. 1,3-Oxazoles can be easily transformed into isocyanide esters **71** by ring-opening with *n*-BuLi and acylating the hydroxylic group formed (Scheme 32) [113]. The odours of **71** depend on the acyl group, but are all likable.

### 3.4. Domino Reactions

Many MCRs are rather tolerant of most other functional groups and these may further react with the newly generated functions of the MCR product in a reaction cascade without additional operations. Thus a high degree of diversity can be achieved. Syntheses in sub-sections 2.14 [64], 2.15 [66], 2.18 [75], 2.19 [77], and 2.20 [84] contain domino reactions. Finally each MCR itself is a domino reaction. For nomenclature see sub-section 1.2.

### 3.5. Post-Condensation-Cyclizations (PCCs)

The reasons in sub-section 3.4 make MCRs also appropriate for a post-condensation-cyclization (PCC), which can build up a cyclisation reaction using the structure generated by the MCR. The PCC can be a domino reaction. Syntheses in sub-sections 2.10 [53], 2.11 [54], 2.14 [64], 2.15 [66], 2.18 [75], 2.19 [77], 2.20 [84], and 2.25 [93] contain PCCs. 

### 3.6. Macrocyclization

The efficient access to macrocyclic structures is still rather difficult. Recent research in this field deals with MCR syntheses of macrocycles, on the approach “multiple multicomponent reaction using two bifunctional building blocks (MiBs)” [114,115].

## 4. New Methods in Performing Conditions to Modern Requirements

Many of the developments of the MCRs in Section 2 are impelled by modern requirements of green chemistry as using water or ionic liquids as solvents or applying solvent-less syntheses and running the reactions at r.t. as well as employing microwave, infrared, or ultrasound irradiation energy in the syntheses. 

### 4.1. Water as Solvent

Most MCRs are tolerant of most reaction conditions and non-involved functional groups, so often water, which does not pollute the environment, can be used as solvent, as in sub-sections 2.9 [52], 2.15 [68], and 2.19 [78,80]. A review on this topic is given [116].

### 4.2. Ionic Liquids as Solvent

The main disadvantage of common solvents is their high vapor pressure, so that losses in the course of a synthesis can be quite substantial and this leakage may pollute the environment. Solvents with a very low vapor pressure could solve the problem. Two kinds of solvents have been proven, ionic liquids (ILs) [117] and long-chained polyethylene glycols. Both have been employed in syntheses of heterocyclic compounds by MCRs. Some syntheses in IL as solvent are described in sub-sections 2.11 [58], and 2.19 [81,82].

### 4.3. Solvent-Less Syntheses

From the ecological and economical points of view, it might be advantageous to run MCRs without any solvent. This will be of course supported by a high ratio of liquid components and their suitable properties. Several syntheses have been carried out with neat components, and detailed studies on this issue have been done in sub-sections 2.4 [33,34], 2.11 [59], 2.17 [74], 2.19 [79], and 2.24 [92].

### 4.4. Alternative Forms of Energy: Microwave, Infrared, Ultrasound Irradiation

Conventional thermal heat as a source of the required energy to bring reactions to run is generally and always employable, but this heat is not selective at all. Certain vibrations of bonds in common compounds, however, can be activated selectively and thus less energy input can achieve the same effect as by using conventional heat.

Water, water-containing and similar species with bridged O-H-O or N-H-N bonds can be activated optimally by microwave irradiation (MW). Species containing C=O bonds absorb infrared irradiation (IR) very strongly. Reactions wherein generated volatile compounds have to be transferred into the gaseous state by evaporation can be essentially supported by ultrasound.

Several MCRs in this paper have been assisted by energy from MW, as in sub-sections 2.8 [47], 2.11 [58,59], 2.15 [66], 2.19 [78], 2.24 [92], 3.1.2 [96], and in [56].

In the 3CR solvent-less synthesis of 6-aryl-1,2,4,5-tetrazinane-3-thione in sub-section 2.24 reaction times and product yields of both conventional heated and MW assisted reactions have been compared with each other [92]. The efficiency of MW on the reactions has been enormous: reaction times have been reduced to 1/15, whereas the yields doubled concurrently. This should be caused by highly polar reactants. A different effect has been observed in a 4CR synthesizing polyaryl-substituted imidazoles in the ionic liquid butylmethyl imidazolium bromide. Reaction time with heat has been half that of with MW, and yields have been similar in both cases [58]. Here the reactants have been highly non-polar. Reviews on the usage of MW in syntheses of heterocycles by MCRs are given [118,119,120,121]. An alternative energy source for reaction activation, even without solvents, is IR [122].

A novel and environmentally-friendly method for preparing dihydropyrano[2,3-c]pyrazoles in wateras solvent and under ultrasound irradiation has been developed [52]. Syntheses of pyrazolopyridines have been performed by 3CRs from aldehyde + malodinitrile + 3-aminopyrazole activated with both conventional heat and ultrasound (sub-section 2.19) [83]. Product yields of ten ultrasound activated reactions are on average 60% higher than those of the same, conventionally heated reactions.

## 5. Conclusions

**Figure 1:**
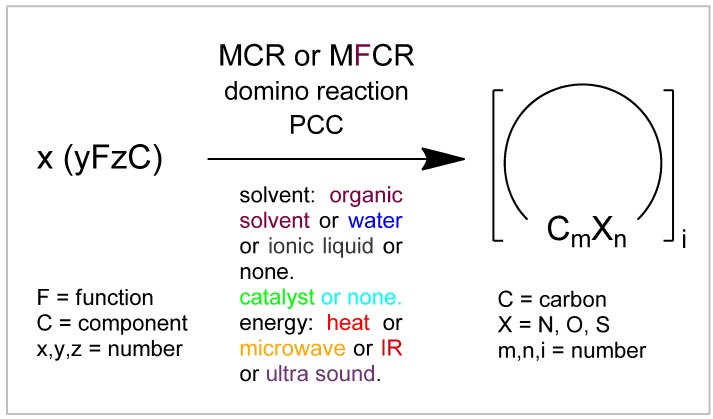
Strategies and tools to design DOS for complex heterocycles by MCRs.

Figure 1 presents a sketch showing the deeper insight of this review, which demonstrates a nearly unlimited up-growth of novel MCRs forming complex heterocyclic structures. Several new smart strategies in combinations of reacting diverse functional groups have been developed and widened the reaction space of MCRs and thus the scope of their application. Particularly reactions of isocyanides with deactivated alkynes such as acetylenedicarboxylates are widely deployable and eagerly investiga-ted, and other smart MCRs are distinctly coming up. But isocyanide-based MCRs still account for a great part of multi-component reactions.

Also several environmentally-friendly new tools have been employed, referring to the use of solvents and energy source. These may offer economical advantages too. Water is useful as solvent for many syntheses by MCRs, because most of their involved functional groups are neutral against water. In special cases solvent-less syntheses provide great advantages from most points of view. 

Often the totally unselective conventional heat can be changed by selective microwave irradiation, which may use less energy. Thereby, components may react much faster, achieving very good product yields. This can also be performed in some cases by use of ultrasonic irradiation. Thus, also in the future further substantial and increasing growth in MCRs for performing heterocycle syntheses is to be expected.
